# Atlanta Academy of Medicine

**Published:** 1876-02

**Authors:** 


### Atlanta Academy of Medicine.

R. W. WESTMORELAND, M.D., REPORTER.

ATLANTA, November 30, 1875.

Dr. McIntosh read the report of a case furnished by Dr. Bailey of Gainesville, in which, after delivery of a child, it was ascertained that the cord had assumed the peculiar form that when put upon the stretch a firm knot would be made.

Dr. J. M. Johnson had witnessed similar cases in his practice.

Dr. J. G. Westmoreland reported a case of labor in which a decoction of gossypium was given, and in which no lochial discharge, to any extent, was found to occur. He asked whether this failure of the usual discharge was probably the result of uterine contraction caused by the uterine excitant given, or a mere coincidence. The labor, which had been slowly progressing for ten or twelve hours, rapidly increased immediately after the remedy was given, and was terminated in less than an hour. That the contractions were increased by the medicine, seemed reasonable to suppose; and therefore, not being in the habit of using cotton-root, would like to know of those who had used it in labor whether they had known such condition to follow.

Dr. John M. Johnson thought there should be very little confidence had in the parturient effects of gossypium or ergot. He has given large quantities of the latter, but had never known contraction of the uterus promoted by it. In answer to a question by Dr. W. F. Westmoreland, as to contraction of the longitudinal fibres, he denies the parturient effect of these remedies entirely.

Dr. W. F. Westmoreland thinks that ergot, in some cases, is sufficiently active in producing contraction of the womb as to result in the death of the child, from the arrest of the placental circulation by the violent, or rather continuous, action thus induced.

Dr. J. G. Westmoreland had repeatedly witnessed what he supposed to be the exciting influence of ergot upon the uterus, in the increased contractions and rapid expulsion of the fœtus. He thinks the longitudinal fibres are particularly acted on, so that the os is made to dilate more readily. He had thought for a long time that the lochia is not necessary to the well-doing of a lying-in female, and the case just reported gives strong proof of the correctness of that opinion. There was, in this case, not only an absence of discharge immediately after delivery, but for two or three days the patient was so entirely free from hemorrhage that the mother and other female friends were so alarmed at the unusual condition that advice was sought. No fever, pain, or other unpleasant symptom was present; and the patient, now two weeks since delivery, is unusually sprightly and cheerful. The general opinion indulged by females, and perhaps some physicians, that puerperal hysteritis and peritonitis result from suppression of the lochial discharge, is erroneous. The inflammatory condition set up by torpid bowels, exciting food, etc., is not the result, but the cause of suppression.

Dr. J. M. Johnson, in answer to the question, why the lochia was necessary, said that the completion of gestation leaves an amount of blood not appropriated, and it should be discharged. He thought the discharge necessary, and that the case reported was an exception to the general rule that a failure proves injurious. He did not think that the cotton-root led to the condition; believed that warm water injected into the bowels and vagina would promote contraction of the womb better than ergot or cotton-root.

Dr. W. F. Westmoreland presented a patient, a boy four years old, with a large lobulated tumor proceeding from the left parotid gland. The tumor had been removed a year ago. It gradually commenced returning, and for the last month has rapidly enlarged, until now it is the size of a man's fist. A short time since he injected a few drops of iodine into one of the lobules, with the view of testing the probability of lessening its size, but finds the increase equally rapid since the injection. He terms the tumor lymphoma. He fears that the pressure upon the trachea, which is now very annoying, will eventually produce death by asphyxia. He proposes to operate by removing a portion of the mass if the shock is too great to remove it all at once.

Dr. Baird thought an operation advisable. The disease will certainly prove fatal without removal, and nothing can be lost by an attempt at relief.

Dr. Calhoun thought an attempt at complete removal would prove unavailing, as portions would necessarily be left, which would make nuclei for the rapid formation of the tumor. He remarked that his information is that experiments prove that the injection of iodine is rarely beneficial, and often injurious.

Dr. J. M. Johnson had had no experience in the removal of such tumors, and did not wish any; but agreed with Dr. Baird in thinking it better to give the patient the only chance of life—an operation.

Dr. J. G. Westmoreland is not at all satisfied that an operation will be of permanent benefit, but thinks its removal, or partial removal, may prolong life. Death is certain without, and probably with, an operation.

Dr. W. F. Westmoreland reported an operation on a man seventy-seven years of age, for strangulated inguinal hernia. He called attention to this case on account of the extreme age of the patient. Failing to reduce the hernia by any means, an operation was determined upon, even after the extreme symptoms of stercoraceous vomiting. Found adhesion, which made reduction difficult. A strange feature in the case was that the bowels moved before the operation was completed; no undue excitement ensued upon the operation, but a few days after the symptoms indicating strangulation, such as stercoraceous vomiting, recurred. The patient has entirely recovered, notwithstanding his age and other difficulties attending his case.

---

JAMES B. BAIRD, M.D., Reporter.

---

Atlanta, December 14, 1875.

Dr. John M. Johnson, President, presiding.

Dr. John G. Westmoreland reported the case of a man æt. about forty years, married, who applied to him about ten days ago, complaining of pain during the act of micturition, and a slight mucus-like discharge from the urethra. The pain was most severe at the vesical neck, and extended to within one inch of the meatus. Cold injections and ice to the penis had been used before he saw him, with the effect of checking the discharge but considerably augmenting the pain. Nitre and other diluents failed to alleviate the trouble. Then injected into the urethra a solution of morphine in gum water, and sulphate of cadmium one grain, to mucilage one ounce. A slight improvement followed this treatment for a few days. Saturday night introduced a catheter, and injected through it the cadmium solution, into the bladder and into the urethra as the catheter was withdrawn. Intense pain followed this treatment, continuing for an hour and a half, and was only relieved by the free use of morphine. Sunday he was almost entirely relieved, but returned to-day (Tuesday) with a recurrence of all the symptoms. There is spasm of the neck, as shown by sudden stoppage of the flow of urine during micturition.

Dr. Todd had recently read a report from a distinguished English writer, recommending an injection of nitrate of silver, ten grains to the ounce, in irritation of the neck of the bladder. Concurs with the writer in thinking the injections ordinarily employed too weak. Does not approve of strong injections into the urethra, but the chloride of sodium contained in the urine neutralized the excess of nitrate of silver when injected into the bladder.

Dr. John M. Johnson thinks Dr. Westmoreland's case one of gonorrhœa. Observed that the pain became greater as the discharge became less. As to treatment, advised frequent hip baths of warm water, opium freely, copaiba and turpentine to expand the urine, and confinement to bed. Thinks no case ever gets well with the patient on his feet. The bowels should be freely purged and an injection of a solution of acetate of lead and sulphate of zinc given, which forms a precipitate, and consequently a protecting layer over the surface of the urethra. Under this treatment his cases get well in four days. In regard to the use of nitrate of silver, has used it from ten to twenty grains to the ounce, and does not think he has ever done harm by it. Thinks its use renders the inflamed mucous membrane insensible to the irritation otherwise produced by the passage of the urine through the urethra.

Dr. Baird reported a case of hemi-chorea in a girl aged 12 years, for the purpose of directing attention to the treatment, the results of which he regards as very satisfactory. The mother stated that she lost a sister several years ago with chorea, and was naturally very much alarmed when choreaic symptoms presented themselves in her child. The cause of the attack was supposed to be a severe fright after excessive fatigue. The mental vigor of the girl was decidedly impaired. There were neither illusions, hallucinations nor delusions, though the sleep was disturbed by disagreeable dreams. The irregular muscular movements were limited to one lateral half of the body, and were frequently so violent in the lower extremity as to throw her from her feet in the attempt to walk. She was unable to feed herself, or to perform any connected series of muscular movements. The muscles of speech and respiration were involved, both of these functions being performed at times with great difficulty. Digestion was imperfect, the bowels constipated, lips pale, and pupils dilated. The disease was gradually progressing when it came under his observation, and would doubtless have invaded the other side if it had not been arrested. The treatment consisted, besides the hygienic measures indicated, of small doses of strychnia—one-forty-eighth of a grain—and sulphate of iron, internally, and ether spray to the spine. The mode of employing the ether is to expose the spine from the occiput to the coccyx, and play a continuous stream of spray, from an ordinary spray apparatus, upon the whole length of the spine, daily, for fifteen minutes. Improvement commenced the day that this treatment was inaugurated. After a few days the supply of ether was exhausted, and its use was omitted for several days, when all of the symptoms increased in gravity. After about twenty-five applications the patient was dismissed, cured.

Dr. J. G. Westmoreland inquired how the action of ether spray was explained.

Dr. Baird replied that he considered its action two-fold: first, the sedative effect upon the spinal cord, from the intense cold produced; and secondly, the reaction acting as a mild counter-irritant.

Dr. Todd asked why he gave strychnia.

Dr. Baird answered, as a nerve tonic. Believes irregular muscular movements frequently the result of debility or exhaustion of the nerve centres.

Dr. John M. Johnson had recently treated a case of chorea for four months; used everything he ever heard of—cold to the spine, tartar emetic ointment, and croton oil, until they ceased to produce the least effect upon the skin; Calabar bean, extract of hemlock, and ether spray. Saw no good from anything, unless it was valerianate of zinc. Thinks the ether spray acts as a revulsive and counter-irritant. Expected great benefit from it when he first commenced its use, but was disappointed. The patient, a young lady aged about sixteen, finally recovered.

Dr. Scott reported the case of a mulatto boy, aged fourteen, that came under his observation six months before, for an injury resulting in dislocation of the radius. The dislocation occurred sixty-three days before he applied for aid. The head of the radius was thrown forward and inward, and in that position firmly fixed. Motion at the elbow slight; supination prevented. He succeeded in reducing it by using considerable force; a splint was applied for a few days. The boy made a complete recovery. Thinks the case of interest from the length of time between the reception of the injury and the reduction of the dislocation.

Dr. Calhoun said that several weeks ago he reported four cases in which he had extirpated the eye-ball for different causes, and he had three more to add to the list. The first, a young lady, æt. twenty; received in early childhood a straight cut in the eye. Soon afterward she was attacked with hooping-cough, and the straining incident to that affection produced a bulging of the contents of the ball at the point of injury, resulting in an immense staphyloma. She also had choroidal inflammation. The ball was extirpated, and she made a speedy and good recovery.

The second case, a gentleman aged fifty years. Four or five years ago observed a pimple on the lower lid, at the junction of the mucous membrane and the skin. This was cauterized, but grew very rapidly. The induration extended under the ball, and involved a portion of the ball itself. It was clearly epithelioma. Vision in this eye was perfect, except the obstruction offered by the contracted lid. He removed the entire lower lid and extirpated the ball. The patient did well, and soon recovered. No effort was made at the time to replace the lid by a plastic operation.

The third was the case of a negro man, who had syphilis several years ago. Eight months ago he treated him for syphilitic irritis in the right eye (iritis gummosa.) He recovered, but several months later he was attacked with complete paralysis, and vision in both eyes was lost. He gradually improved under anti-syphilitic treatment. The right leg is not yet entirely restored, and his voice is not clear. Sight was not restored to the right eye, and it was the seat of great pain. He discovered pus in the ball, and a small portion passed through the pupil into the anterior chamber. The cornea was punctured, and the pus from the anterior chamber evacuated. Two small tumors subsequently appeared upon the upper part of the sclerotic. The other eye soon became affected. The right ball was extripated, and afforded immediate relief. Recovered.

He reported another case in which it will be necessary soon to remove the ball. A child, ten months old—glioma (cancerous tumor) of the retina. The child is vigorous and well nourished. Five months ago the mother noticed a white body in the pupil. It continued to grow until sight was destroyed. A few days ago a similar growth was discovered in the other eye. The ophthalmoscope shows the tumor attached to the retina, and blood vessels running over the surface. Sight will soon be lost in this eye. The remedy—a terrible one—consists in extirpating both balls. If the disease is not checked the cornea, which is already beginning to soften, will break through and the tumor spread over the face. The disease will also extend along the optic nerve, and soon the brain will become involved, and death speedily follow. In answer to an inquiry, Dr. Calhoun stated that the disease was apt to return unless the whole of the diseased tissue was removed. Adjourned.

---

R. W. WESTMORELAND, M.D., REPORTER.

---

ATLANTA, December 21, 1875.

Meeting called to order by the President, Dr. John M. Johnson.

Dr. J. G. Westmoreland, in reference to the case of acute urethritis reported at last meeting, reported improvement. Remarked that the neck of the bladder was still inflamed, causing almost constant desire to urinate.

Dr. Logan, by request of Dr. Todd, referred to the case of accidental poisoning, by opium, of a child, in which veratrum was administered for the purpose of quieting the cerebral excitement existing prior to the poisoning. He remarked that no further opportunity had presented itself for investigating the subject.

Adjourned.

---

ATLANTA, December 28, 1875.

Meeting called to order, Vice-President John M. Boring in the chair.

Dr. J. G. Westmoreland reported a case of supposed impaction of the cecum from imperfectly masticated locust-hulls. Retention of urine was a very strange feature in the case. One gallon of water was injected into bowel without perceptible benefit. This was repeated in three or four hours, with some increase in the quantity, and with good results—a healthy action being brought about. Reported, in this connection, a case in which stercoraceous vomiting had commenced from intestinal obstruction, and in which an injection of considerable amount of water gave perfect relief. Reported also a case of obstruction from plumb-hulls, in which, after a large quantity of water had been injected, no fecal odor was discovered on its discharge, but a second injection produced an operation and relief. Called attention to the fact that often large quantities of water at first bring no fecal odor. Would like to know the cause of such phenomenon.

Dr. Scott reported a case of intestinal obstruction where there had been no operation for six days. A diet of light bread had produced the obstruction. One gallon of water was injected without bringing any fecal odor. A second injection gave relief. Does not think that this or the preceding cases were impaction of cecum, as it does not seem probable that water could be in contact with the impacted mass without taking the odor. Discussion as to the seat of the odor-generating apparatus, was entered into, and also as to the probability of impacted cecum in the preceding cases, by Drs. J. G. Westmoreland and Scott.

Dr. Baird mentioned a case of obstructed bowel in which the breath had a stercoraceous odor, while repeated injections of large quantities of water returned without any odor. This would seem to indicate the possibility of the small intestine being the source of the fecal odor.

Dr. J. G. Westmoreland gave it as his opinion that the small intestines created the odor, as the case just reported would imply.

Dr. Taliaferro reported a case of a double uterus in a young unmarried lady of twenty years. Distinct, each, in all their parts, measuring two and a quarter inches in depth. The lady has some endometritis for which she came to be treated, not enough, however, to give much trouble. A collection or tumor in Douglas' *cul-de-sac*, is the principal point to which his treatment is directed.

Dr. Armstrong having seen the above case with Dr. Taliaferro, gave it as his opinion that it was a case of complete double uterus. Each uterus is natural in appearance, so far as he could see; lying parallel to each other, and seemingly in normal situation. On inquiry as to size, he thought both together was larger than a normal uterus.

Dr. Taliaferro remarked that each cervix was as large as that of normal uterus.

Dr. Boring was anxious to know what would be the result of impregnation.

Dr. Armstrong asked to know if there were any members who had seen the like. None had been seen. Adjourned.

---

### VERATRUM AN ANTIDOTE TO OPIUM.

BY J. S. TODD, M.D., one of the Vice-Presidents.

*Read before the Atlanta Academy of Medicine.*

This assertion is the result of the fact that opium is an antidote to veratrum. That it is, none who have had any experience with overdoses of veratrum will deny. Dr. Norwood, to whom we are mainly indebted for this valuable drug, recites several cases where large quantities of his tincture were given; in one instance as much as one drachm, which caused the most alarming prostration, vomiting and general relaxation; all of which symptoms were almost immediately dispelled by opium and whisky. I have in my own practice had frequent occasions to verify his statement, that there need be no fear of a fatal result from veratrum if the antidote above mentioned is given. Indeed, the antagonism is as marked as that of acids and alkalies. The question very naturally resolves itself into this shape: are antidotes reciprocal? The answer is obvious; it cannot be otherwise but in the affirmative; for an acid is no more incompatible with an alkali than an alkali with an acid. Belladonna, or its alkaloid, atropia, has for some time enjoyed the confidence of many of the most learned and distinguished in our profession as an antidote to opium. It is not my object or intention, in this paper, to attempt to prove that it is not an antidote; for, with Prof. Reese of Philadelphia, I am constrained to acknowledge, that after a fair and honest review of all the cases, pro and con, as to the antagonism of belladonna and opium, the evidence in their favor has preponderance. This testimony I give, in the face of the fact that experiments on the lower animals seem to show that where the two are given together, the effect of each is intensified. Dr. Harley, F.R.C.P., F.L.S., etc., in the Galstonian Lectures of 1868, extended and including a complete examination of the active constituents of opium, arrives at a totally different conclusion. In this lecture the question of the antidotal action of opium and belladonna are considered at great length. The recorded cases are carefully examined and classified in three tables: the analysis of these, in connection with very many and varied experiments, "justifies the conclusions that the evidences of antagonism are inconclusive; that belladonna has no influence whatever in accelerating recovery from the poisonous effects of opium; but on the other hand, the effects of the opium are intensified and increased. It is utterly powerless in obviating the chief danger of opium poisoning, the depression of the respiratory function." He states, however, that in doses of from the 1-96 to the 1-100 of a grain, atropia is the greatest and most potent of all agents, not excepting carbonate ammonia and whisky, as a cardiac stimulus, and that in these doses it may, and doubtless will, do good, where the pulse is very slow or weak. How much atropia is necessary to counteract a given quantity of morphine, has never been ascertained. We all give it with fear and trembling, knowing that the remedy, if pushed, is as bad as the disease. In other words, the remedy is too dangerous. If any of us were called to a case where an alkali had been given in hurtful dose, we would not administer nitric, chromic or muriatic acids, but rather vinegar or citric acid; something that would not destroy both the poison and patient. I propose as a substitute for the too potent deadly night-shade, the milder veratrum. Beck, in his work on Medical Jurisprudence, classes veratrum and belladonna under the same head—acrid narcotics—both dilating the pupil and killing by producing convulsions. There would seem to be, from this, some analogy between their action in poisonous doses. It is only by careful scrutiny of recorded observations as to the effects of drugs that any valuable contribution to therapeutics is obtained. With this view I present the following cases, the notes of which were carefully and truthfully taken at the time. As to the *modus operandi*, I do not attempt to explain it. The very best reason that can be given why we administer this or that is, *it is successful.*

J. H., æt. thirty, dissipated, took, October 26, 1872, in my presence, little over six drachms of tinct. opii. Assuming thirty drops of laudanum to be equal to a grain of crude opium, he took at one dose twenty-four grains. He had taken half an ounce of laudanum ten or fifteen minutes before this, (16 grains) thus taking in all forty grains of opium, or 1,200 drops of the tincture. All persuasion to induce him to take an emetic being futile, I prevailed on several bystanders to throw him down, and then vainly attempted to give him a mixture of ipecac and sulphate of zinc aa. grs. xxx. I could not make him swallow, though his nose was held and threats used, until I poured some of the solution in his eyes, which caused so much pain that he came to terms, and took the emetic a few moments after. It not having the slightest effect, a drachm more of ipecac was administered. In ten minutes he was asleep. I had him walked and shaken, thinking that the emetic would act could he be kept awake. There was no stomach pump in the place. He soon ceased to step or exhibit any symptom of pain or sensibility whatever. Placed his head under a pump, and gave him the cold douche five minutes. Meanwhile the coma gradually and fearfully increased. Had him carried to Dr. McMillan's office, where there was an electric battery. I was here joined and assisted by Dr. Henderson. Under the electric stimulus he so far regained consciousness that Dr. McMillan and myself concluded to give him more ipecac. This was 8 o'clock. Suddenly, while taking it, he collapsed, and some of the fluid passed down the trachea. Respiration was kept up partly by artificial means, but principally by placing one pole of the battery along the course of the pneumogastric nerve, above the sternum, and the other at the pit of the stomach, for at least three quarters of an hour. At this juncture the glass of the battery containing the acid broke, and we had no substitute. Mustard was applied to the spine, chest and abdomen, and friction used on extremities. Pulse slow, full, 30; respiration stertorous, 8; face much congested; nose and ears purple; extremities cold. Had him moved to his room, sent for an old-fashioned friction magnetic battery, the only one in the town. The pupils were, of course, tightly contracted. We concluded to try hypodermic injection of atropia; it was administered until the pupils were widely dilated. At 11 P.M. Drs. McM. and H. left me, saying the man would die very soon; such was also my opinion, but I determined to give him every attention. The battery was used on him with occasional intermissions until three o'clock, when his breathing was six, and the pulse suddenly ceased. I immediately injected under the skin a drachm of raw whisky, and felt the pulse return before the syringe was withdrawn. After his pupils were dilated by the atropia his pulse became rapid and weak. Fifteen minutes later I injected another drachm of whisky and two drops of tincture of veratrum viride. Five minutes after the dose his pulse became slower, but weaker, and there was paralysis of the entire right lung. No air whatever entered or was expelled from it for two or three respirations. I now had him violently rolled about the bed, slapping and compressing the chest. Five or six such turns caused a groan and an emesis of four or five ounces. He opened his eyes, but immediately closed them and relapsed into the comatose condition. This was at 3:15 A.M., just fifteen minutes after the first dose of veratrum. From that time until 5 I gave him six hypodermic injections of a drachm each of raw whisky; and two, an hour apart, of two drops each of tinct. veratrum viride, with marked improvement each time. He was meanwhile persistently and continually worried, the battery applied to spine, the cold douche was used, and he was walked, slapped, whipped, tickled, etc. Any such procedure, however, after two or three repetitions, lost all effect. By 5 A.M. he was able to swallow a teaspoonful of very strong coffee, one cup of grounds to three of water. By 8 he had taken four or five of such cups, and vomited freely. After this he vomited several times, but at 9 he retained a tolerably stiff drink. Once, about half-past 6, I let him take a nap. Whenever he was not worried he slept, and would, while sleeping, have general convulsive movements, and the breathing would fail from 14 to 8 per minute. I did not allow him to lie down at all during the day, although he was pretty thoroughly aroused by 10 A.M., and perfectly rational. I permitted him to take naps of from ten to thirty minutes, sitting. He vomited everything he took that day, except the toddy before mentioned.

October 28th.—Slept tolerably well last night, but was very nervous.

November 1.—Has suffered considerably from nervousness and irritability of the stomach during the past three days. Gave laudanum, bromide of potassium and quinia, *pro re nata.*

On October 29th he left for his home, and November 2d he resumed his work, The whisky did not cause a single abscess; all soreness about the chest, where injections were made, was superficial, and plainly attributed to the mustard, which blistered in several places over the thorax, and the skin is still highly inflamed wherever it was applied. He has complained always of his throat—described the sensation as brassy, and was, until to-day, hoarse. This is doubtless caused by atropia. His bowels moved spontaneously on the day after he took the laudanum.

November 12th.—Been at work steadily since the 2d. No abscess has developed itself.

Remarks.—Atropia in this case certainly did no good; the veratrum much. In fact, to it do I mainly attribute the recovery, but I do not believe it would have been sufficient in itself. My timidity in the use of the agent doubtless put off the good effect. This man was as persistently worried as three of the most obedient attendants could possibly perform such an office. They kept him alive from the combined effects of both drugs, until the veratrum suggested itself. It will be particularly noticed that no emesis occurred until veratrum viride was given.

Case II. December 30, 1873.—B. R., æt. fifty-two, merchant. Had suffered from occasional mental derangements for eighteen months. During such attacks he attempted to take his life in various ways, by drowning, the razor, and phosphorous. To-day he procured from J. P. M. & Co., druggists, an ounce of gum opium, and before he was detected he had eaten the whole of it. This he did about an hour before I saw him. When I first saw him, he was highly intoxicated from it, covered with a profuse perspiration, and somewhat sleepy. He persistently refused to take anything; was perfectly rational. About half an hour was lost in persuasion and force, endeavoring to get him to take zinc and ipecac; fully another half hour, in having a funnel made to fit the end of a catheter (male). Through it, introduced down the nose into the pharynx, I gave sixty or seventy grains sulph. zinc, and as much ipecac. He almost immediately vomited. In the emesis there was much opium; the smell of it could be detected diffused over the room. During all this time he was of course worried and walked, but the coma continued to increase. I then gave him every half hour, ten to twelve drops of tincture veratrum veride, hypodermically, with an emesis, or an attempt at vomiting after each injection of veratrum. Everything not given by hypodermic means had to be given through the catheter inserted in the nose down the throat. He took the opium about 11½ o'clock A.M. Until 6½ his pupils did not contract, nor did he become so drowsy that he ceased to step, or give rational answers, or evince pain when flagellated, or when the battery was put to him; nor did his pulse become slow, or respiration stertorous. About this time Dr. Griggs, family physician, came, and only one more dose of veratrum was given, Dr. G. having no confidence or experience in the veratrum treatment. He recognized Dr. G., and spoke to him. His pulse at that time was was 65; respiration 15 or 16. We commenced to use the cold douche, and at 7 o'clock gave atropia, which undoubtedly increased the coma. We gave it until the pupils were dilated, for they began to contract at 6. Mustard was applied to chest, abdomen, spine, and extremities. His pulse never became *slow nor full*, but his breathing did after the atropia was given. At 8 o'clock exactly, he died suddenly, from asphyxia, the heart continuing to act for several minutes after respiration ceased. Artificial respiration could not be induced with the battery.

Remarks.—The large amount of opium, and the length of time that elapsed between the taking and commencement of treatment, are sufficient to convince any thinking man that there was but one termination. The good effects of the treatment are manifest by the length of time that intervened before death. Not only was life prolonged, but the usual effects of opium were long in showing themselves. According to Dr. Christison, the usual duration of fatal cases is seven hours. This patient took hypodermically, in less than six hours, a drachm of tincture veratrum viride without lowering the pulse; in fact, it seemed to keep the heart going.

Case III. February 2d, 1873.—Was called, in consultation with Dr. McMillan, to see W., æt. 12, who was suffering with meningitis. We concluded to give morphine. In fifteen minutes the symptoms were characteristic of opium poisoning: contracted pupils, no pulse, stertorous breathing, and coma. Under the cold douche and three injections of three drops each of tincture veratrum viride, he promptly vomited, pulse returned and pupils dilated in from one and a half to two hours. The morphine was given about 11 A.M. He died at 11 P.M., from the force of the disease, with no symptoms of opium.

Remarks.—The death of the patient in no way invalidates the conclusion that I am trying to prove. The epidemic of meningitis, which carried off this boy, was very fatal. The return of the pulse and dilatation of the pupils from the effects of the veratrum are particularly worthy of notice.

Case IV. December 23d, 1875.—C., child six weeks old. Had taken five drops of a tincture of opium, which had remained unstopped until of a syrupy consistency. The dose was taken at 4 o'clock P.M. I saw it first at 6 P.M.; the breathing was 12, and sighing; pulse 60; coma well marked; pupils tightly contracted. By vigorous flagellation, or rude handling, could be induced to open the eyes, but efforts to make it swallow anything were futile. Gave, hypodermically, one drop of tincture veratrum viride, in 10 of brandy. One hour afterwards repeated the dose. In fifteen minutes it vomited slightly, about half an ounce of mucus. At 8½ there was another slight emesis; the symptoms unchanged, except more mucus in lungs. The cold douche was used, but produced no evidences of sensibility. Injected from four to six ounces of strong coffee per anum, which was partially retained. This was repeated about 9. At 9½ injected one-half drop of veratrum and fifteen of whisky. At 10¼ the child vomited freely, copiously, with instant improvement in every symptom. During the vomiting the coffee was passed from the bowels. It would cry and exhibit evidences of pain from slight inflictions, or even from loud speaking. At 10½, respiration 24, pulse 100; pupils normal. Mustard was used to spine and abdomen.

December 24th.—Except from soreness, the child was in usual health.

Remarks.—Half an hour before the last emesis the child was excessively nauseated and much relaxed. How much opium there was in the five drops this infant took is not certain, but it is a well-known fact that alcohol, and not the opium, is the constituent which evaporates. It must have taken at least eight drops of the ordinary tincture. Was this amount sufficient to cause death?

Dr. Kelso, in the *London Lancet*, vol. 21, page 304, relates a case where four drops of laudanum to a child thirty-six weeks old was fatal. A case mentioned in the *Medical Times*, vol. 10, page 436, where two drops of the tincture, given four times during eighteen hours, killed a child six weeks old. The cure in this case is to be attributed to the veratrum alone. Improvement in the pulse, after vomiting, is especially noteworthy.

CASE V.—Dr. E. H. Sholl, of Alabama, communicated to the Philadelphia *Medical and Surgical Reporter* a case of poisoning by morphine, which was cured by veratrum. The patient a negro boy fifteen years old, took an overdose of morphine, which had been prescribed for hiccough. It was followed by stertorous breathing, contracted pupils, etc. Eighteen drops tincture veratrum, with two ounces of brandy, after one hour, caused all the symptoms to vanish.

CASE VI.—Dr. J. P. Logan, of this city, related before the Academy a case of opium poisoning, in which he gave tincture veratrum to control the cerebral symptoms. The result was an entire cure.

While I feel every confidence in veratrum as an antidote to opium, I would by no means neglect, under all circumstances, to give strong coffee when the patient could swallow it, and when he could not be induced to take it per orem, I should give it per anum, and also place particular stress on having the unfortunate walked, flagellated, tickled, etc. Obedient, active attendants are a host in themselves, and in no other emergency does the physician need such allies more. Electricity is also an agent not to be neglected. Of course no one will neglect to use every means to evacuate the contents of the stomach.

